# Amino acid composition of plant protein-enriched wheat biscuits differentially affects postprandial amino acid responses of overweight/obese compared to normalweight subjects

**DOI:** 10.1007/s00394-025-03759-x

**Published:** 2025-07-21

**Authors:** Amalia E. Yanni, Maria-Christina Kanata, Varvara Papaioannou, Maria Halabalaki, Vaios T. Karathanos

**Affiliations:** 1 Laboratory of Chemistry-Biochemistry-Physical Chemistry of Foods, Department of Nutrition and Dietetics, Harokopio University, 17671 Athens, Greece; 2 Division of Pharmacognosy and Natural Products Chemistry, Department of Pharmacy, National and Kapodistrian University of Athens, 15771 Athens, Greece

**Keywords:** Amino acids, Plant protein, Biscuits, Snacks, Postprandial responses, Overweight/obesity

## Abstract

**Purpose:**

The study investigates whether postprandial amino acid responses differ between normalweight (NW) and overweight/obese (OW) individuals following consumption of plant protein-enriched wheat biscuits with the same protein content but different protein composition. It highlights the importance of developing functional snack products with specific amino acid profile that could benefit individuals with overweight/obesity.

**Methods:**

Thirty volunteers (15 NW and 15 OW) participated in an acute, randomized crossover trial, in which they consumed two plant protein–enriched wheat biscuits differing in amino acid profile—one enriched in L-arginine (arginine biscuit, ArgB) and the other in branched-chain amino acids (branched-chain amino acids biscuit, BCAAsB)—as well as a conventional wheat biscuit (CB) in separate sessions with one week intervals. Postprandial amino acids (AAs) responses were measured for 180 min following ingestion. Fasting and postprandial AAs concentrations were determined by Ultra-High Performance Liquid Chromatography coupled with Time-of-Flight Mass Spectrometry (UHPLC-ToF–MS).

**Results:**

OW subjects exhibited higher fasting concentrations of methionine, tryptophan and tyrosine (*p* < 0.05) while NW subjects, higher levels of glutamine (*p* < 0.05). Postprandial responses of all the determined AAs to enriched biscuits were higher in the NW compared to the OW group with incremental areas under the curve (iAUCs) of alanine, glutamine and threonine reaching statistical significance (*p* < 0.05). In the OW group, ingestion of BCAAsB resulted also in lower iAUC values of asparagine and serine, while consumption of ArgB led to a lower iAUC of glycine and a higher iAUC of taurine compared to the NW group (*p* < 0.05).

**Conclusion:**

Ingestion of plant protein-enriched wheat biscuits by OW subjects resulted in lower postprandial responses of alanine, glutamine and threonine compared to NW. AAs composition of BCAAs-enriched biscuit resulted also in lower responses of asparagine and serine while that of the L-arg-enriched biscuit, in lower glycine but higher taurine responses in OW compared to NW subjects. Depletion of all the above-mentioned AAs has been recorded in obesity. Higher taurine concentrations can lead in beneficial effects since taurine improves glycemic control and insulin sensitivity and it has shown potential anti-obesity properties. These findings underscore the challenge of designing protein-rich foods that could elicit beneficial metabolic responses for overweight/obese individuals.

## Introduction

Plant proteins have been linked with a broad spectrum of positive health outcomes, particularly in the context of chronic disease prevention. Substituting part of animal-derived proteins with plant-proteins has been associated with improved lipid profile, including lower levels of LDL-cholesterol and apolipoprotein B, enhanced glycemic regulation, such as lower fasting glucose and insulin levels and reduction of daily energy intake [1]. These changes contribute to a lower risk for developing cardiovascular disease (CVD), type 2 diabetes mellitus (T2DM), obesity and metabolic syndrome [2, 3]. Incorporating plant proteins, particularly those derived from legumes and seeds, into popular, appealing, and convenient snacks can be an effective strategy for enhancing their nutritional profile.

The alarming increase of global obesity rates has become a major health concern over the recent decades [4, 5]. Obesity results from a chronic positive energy balance, that progressively leads to increased fat accumulation in the adipose tissue. The condition of obesity is strongly correlated with insulin resistance (IR), a key predictor of pre-diabetes and risk factor for T2DM [6]. It causes alterations in protein metabolism due to changes that induces in insulin secretion and utilization of amino acids (AAs) from dietary proteins. IR causes a blunted protein anabolic response, leading to higher protein breakdown and elevated fasting AAs concentrations [7–9]. In obesity, adipose tissue becomes metabolically dysregulated and secretes elevated levels of pro-inflammatory cytokines, such as tumor necrosis factor-alpha (TNF-*α*) and interleukin-6 (IL-6), along with altered levels of adipokines including leptin and adiponectin. This contributes to a chronic low-grade inflammatory state that further impairs amino acid metabolism [10]. For instance, circulating glycine levels are often reduced in obesity, which may reflect both increased metabolic demand and impaired biosynthesis, ultimately diminishing glycine’s anti-inflammatory and cytoprotective roles [11].

Obesity is also associated with altered postprandial amino acid metabolism, largely due to IR, which impairs amino acid uptake and utilization. These physiological changes can lead to differences in circulating amino acid profiles and their metabolic fate, even when the same quantity and type of dietary protein is consumed [8, 12–14]. Elevated levels of specific plasma AAs have been correlated with obesity [12, 13, 15, 16]. Branched-chain amino acids (BCAAs) and aromatic amino acids (AAAs), are linked to higher body mass index (BMI) and impaired glucose metabolism [17–19]. Fasting levels of the aforementioned AAs can predict future development of CVD and T2DM, before the rise of clinical markers, such as blood glucose [20]. According to research studies, weight loss, even only at the level of 3% of body weight, can normalize plasma AAs concentrations such as BCAAs, AAAs, alanine and proline [21–23]. Obesity has been correlated with lower circulating concentrations of AAs such as glycine, serine, and glutamine, while higher levels of these AAs have been associated with reduced visceral fat accumulation and improved metabolic profiles [14, 15, 18, 24].

Most studies mainly focus on fasting circulating AAs levels, but the examination of the postprandial AAs responses can evaluate the impact of the different AAs composition of the ingested food in the condition of obesity and IR [20]. Postprandial AAs responses are enhanced after weight loss in subjects with morbid obesity, with changes being even more pronounced compared to the fasting state and can potentially predict changes in glucose, insulin, and incretins like GLP-1 [23]. The effect of AAs composition of an ingested food on the magnitude of AAs postprandial responses of OW compared to ΝW subjects is a field which is worthy of further investigation. As it has been mentioned, specific AAs act as metabolic regulators by influencing insulin secretion, glucose metabolism, and signaling pathways.

Furthermore, the digestibility and absorption kinetics of dietary proteins can have an impact both on the timing and the magnitude of amino acid appearance in the circulation. Therefore, the amino acid composition of a specific food product has the potential to influence postprandial metabolic responses, depending not only on the type of protein consumed but also on the individual’s metabolic state. This effect may be particularly relevant in individuals with metabolic disorders, such as obesity and T2DM, where amino acid handling and insulin sensitivity are often impaired. A previous study by our research group has shown that ingestion of wheat biscuits enriched with plant proteins with different qualitative composition resulted in significantly lower glycemic responses and better subjective appetite ratings compared to a conventional wheat biscuit However, despite containing nearly identical amounts of protein, the different amino acid compositions of the two plant protein-enriched biscuits led to distinct postprandial insulin and gut hormone responses [25]. In the context of this study, two protein-enriched wheat biscuits were developed: one enriched in BCAAs and the other in L-arginine (L-arg)—both of which have been associated with beneficial effects on appetite regulation. Wheat biscuits were chosen as a familiar, widely accepted, and palatable food vehicle for incorporating protein-rich legume and seed flours. Their standardized shape and portion size allow for controlled nutrient delivery, which is essential in postprandial studies [26, 27]. As a commonly consumed snack, they reflect a real-world dietary context, enhancing the translational relevance of our findings.

The plant proteins incorporated into the biscuits, which were sourced from legumes (e.g., white beans, lentils) and seeds (e.g., millet, sesame), were selected based on their naturally high concentrations of BCAAs and L-arg, which have been linked to favorable metabolic outcomes. Leucine, a key BCAA has been shown to stimulate insulin secretion, activate mTOR signaling, promote muscle protein synthesis and glucose regulation, and reduce food intake [28–30]. Arginine facilitates vasodilation and improves glucose uptake in peripheral tissues [31, 32].

The aim of the present study was to evaluate whether consumption of the same high nutritional quality snack i.e., a plant protein-enriched wheat biscuit, would elicit different postprandial AAs responses between NW and OW individuals. Comparing the responses between NW and OW individuals can help clarify how excess body weight influences AAs handling, providing insights for dietary strategies aimed at improving metabolic health. The study also investigates how the different AAs composition of the plant proteins used to enrich wheat biscuits affects individual AAs responses in NW and OW subjects. Finally, the study aimed to highlight the potential of specific AAs compositions to support metabolic regulation, emphasizing the importance of developing functional snack products that could benefit individuals with overweight/obesity.

## Methods

### Test foods

Two types of wheat-based biscuits were developed with equal protein content derived from a mixture of plant flours, which were prepared from a combination of legumes and seeds commonly consumed in the Mediterranean cuisine. The flours were combined to enrich the final products with specific AAs, especially those which are related to appetite regulation i.e., L-arg in ArgB and BCAAs in BCAAsB [25]. A common wheat biscuit (CB) based on the same recipe has been also prepared which contained only wheat flour and served as control. The composition of flours of BCAAsB (final product) was 28.8% wheat flour, 18.9% millet flour, 4.7% white bean flour and 10.5% yellow lentil protein concentrate (which consists of 55% protein on dry basis). The composition of ArgB was 41.2% wheat flour, 11.8% defatted sesame flour and 5.9% yellow lentil flour. CB contained 57.4% wheat flour.

Nitrogen (protein: Nx6.25) was determined by Kjeldahl (ISO 1871:2009) [33] and fat by Soxhlet methods [34]. Total, soluble and insoluble dietary fibers were determined by the AOAC method 991.43 [35]. Available carbohydrates were calculated by difference, using the following equation: Carbohydrates (%) = 100 − (Moisture + Protein + Fat + Ash + Fiber). Energy content (kJ) was calculated according to the following equation: Energy = 17 x (g protein + g carbohydrate) + 37 x (g fat) + 8 x (g total dietary fibers).

Individual AAs were separated by ion exchange chromatography and determined by reaction with ninhydrin using photometric detection (EU 152/2009).

### Study subjects and experimental design

The experimental protocol was conducted in the Diabetes Laboratory of the 1st Department of Propaedeutic and Internal Medicine, Laiko General Hospital, Athens School of Medicine, in collaboration with the Laboratory of Chemistry-Biochemistry-Physical Chemistry of Foods, Department of Nutrition and Dietetics, Harokopio University of Athens, Greece. The study received approval from the Institutional Review Board/Ethics Committee of Laiko General Hospital and the Bioethics Committee of Harokopio University, and it was registered at clinicaltrials.gov with the identification number NCT03974165.

In total, 30 apparently healthy subjects (15 males and 15 females) whose BMI ranged between 19.1 and 34.5 kg/m^2^ participated in the experimental protocol. Specifically, from the total study participants, 15 subjects (8 women and 7 men) were normalweight (NW) with BMI 21.9 ± 1.9 kg/m^2^ and 15 subjects (8 women and 7 men) were classified as overweight/obese (OW) with BMI 29.5 ± 3.0 kg/m^2^ (mean ± SD) [36].

Participants were apparently healthy adults recruited from the general population through poster advertisements and personal communication. Exclusion criteria were current attempts to modify diet or physical activity for weight loss, restrictive eating patterns, known food allergies or intolerances, engagement in excessive exercise or competitive sports, use of medications or dietary supplements, pregnancy, breastfeeding, and postmenopausal status. Eligible participants were instructed to maintain their habitual dietary and physical activity habits and to avoid significant weight fluctuations throughout the study period.

Participants arrived at the study site (Diabetes Laboratory, 1st Department of Propaedeutic and Internal Medicine, Laiko University Hospital) between 08:00 and 09:00 after an overnight fast. Upon arrival, anthropometric and baseline blood pressure measurements were taken. Body weight and body fat percentage were assessed using a calibrated bioelectrical impedance analyzer (Tanita WB-110MA, Tokyo, Japan), height was measured with a stadiometer (Seca Mode 220, Germany), and waist and hip circumferences were measured using standard protocols.

Following a 10 min rest, an intravenous catheter was inserted into a forearm vein for serial blood sampling. Participants were provided with one of the test meals—two plant-protein-enriched biscuits (BCAAsB and ArgB), or a conventional wheat biscuit (CB)—in a randomized order. Each test meal delivered 50 g of available carbohydrates and was consumed within 15 min.

Blood samples were collected before meal ingestion and at 15, 30, 45, 60, 90, 120, and 180 min postprandially. The study followed a single-blind, acute randomized controlled crossover design with a one-week washout period between sessions. To ensure consistency across visits, participants were instructed to consume the same dinner the evening prior to each study session. Randomization of the test meal order was performed using a computer-generated schedule to ensure that each participant received the three test biscuits in a random sequence. Additionally, 24-h dietary recalls and physical activity questionnaires were collected before each visit.

The basal biochemical measurements which were performed to confirm that all participants were within normal ranges prior to study initiation, were conducted using an automated biochemical analyzer (Medilyzer, Medicon Hellas S.A., Athens, Greece) with commercially available diagnostic kits. Insulin was assayed using a sandwich ELISA method (Human Insulin ELISA kit, Merck-Millipore, Burlington, MA, USA) [25]. Plasma samples for AAs determination were stored at − 80 °C until analyses which were performed after the completion of the research protocol. AAs were determined by Ultra-High Performance Liquid Chromatography combined with Time-of-Flight Mass Spectrometry (UHPLC-Triple-TOF) using the aTRAQ® reagent kit [25, 37].

### Calculations and statistical analysis

IR was calculated by the homeostasis model assessment of IR index (HOMA-IR) as follows [38]:

$$\begin{aligned} \begin{aligned} HOMA-IR & = \left( {Fasting\;Plasma\;Glucos e\left( {FPG) (mg/dL)}\right)} \right. \\ & \quad \left. { \times Fasting\;Serum\;Insulin\left( {FSI} \right)\left( {\frac{{\upmu {\text{U}}}}{{{\text{mL}}}}} \right)} \right)\Big/405 \\ \end{aligned} \end{aligned}$$

Insulin secretion was calculated by the homeostasis model assessment of *β*-cell function (HOMA-*β*) as follows [26]:

$$ HOMA - \beta = {{\left[ {{\text{FSI }}(\upmu {\text{U}}/{\text{mL}}){\text{ }} \times {\text{ 36}}0} \right]} \mathord{\left/ {\vphantom {{\left[ {{\text{FSI }}(\upmu {\text{U}}/{\text{mL}}){\text{ }} \times {\text{ 36}}0} \right]} {\left[ {{\text{FPG }}\left( {{\text{mg}}/{\text{dL}}} \right) - {\text{63}}} \right]}}} \right. \kern-\nulldelimiterspace} {\left[ {{\text{FPG }}\left( {{\text{mg}}/{\text{dL}}} \right) - {\text{63}}} \right]}} $$

The results are presented as mean ± SD or mean ± SEM. Fasting plasma AAs concentrations were calculated as the average of the three baseline measurements from each study visit for each participant. Areas under the curve (AUC) of AAs responses were calculated applying the trapezoidal rule using the incremental AUC (iAUC), ignoring area under the baseline [39]. The Kolmogorov–Smirnov test was applied to examine normal distribution of variables. Statistical significance of normally distributed data between groups was examined by independent samples Student’s *t* test. The SPSS 21.0 statistical software package (IBM SPSS Statistics for Windows, Version 22.0, Armonk, NY, USA: IBM Corp) was used for the analysis and* p* < 0.05 was considered statistically significant.

The number of participants was determined based on a power calculation for the primary outcome, which was the change in glucose iAUC over a 180 min postprandial period. Postprandial amino acid concentrations were also measured and were classified as secondary outcomes. However, it is important to note that the study was not specifically powered to detect differences in amino acid responses, and these analyses should be considered exploratory.

## Results

### Test food analysis

The macronutrient composition per 100 g of food as well as per 50 g of available carbohydrates of the two plant protein-enriched biscuits and that of the control biscuit is presented on Table 1. Both BCAAsB and ArgB contained twice the total protein and AAs amount compared to CB. The AAAs content of BCAAsB and ArgB was similar between them, and they were 50% richer compared to the CB. The specific AAs composition of the three biscuit samples per 100 g is presented on Table 2a. Twenty AAs were determined i.e., L-alanine, L-arginine, L-aspartic acid, L-cysteine, L-glutamic acid, glycine, L-histidine, hydroxy-L-proline, L-isoleucine, L-leucine, L-lysine, L-methionine, L-phenylalanine, L-proline, L-serine, L-threonine, L-tryptophan, L-tyrosine, L-valine and L-ornithine. The amounts of AAs per 50 g of available carbohydrates which were consumed, is presented on Table 2b. The energy contents of the three biscuits were 1874.9, 1893.3 and 1873.6 kJ/100 g for BCAAsB, ArgB and CB, respectively.

**Table 1:** Nutrient composition of the three tested biscuits (BCAAsB, ArgB and CB) expressed in g per 100 g of food and in g per 50 g of available carbohydrates [25]

Nutrient	BCAAsB	ArgB	CB
*per 100 g*			
Proteins	14.0 ± 0.7	14.5 ± 0.7	7.3 ± 0.5
Carbohydrates (available)	60.0	56.8	71.5
Sugars	10.0 ± 1.0	12.0 ± 1.0	22.0 ± 2.0
Total fat	15.7 ± 0.3	17.2 ± 0.3	14.0 ± 0.2
Total dietary fibers	4.5 ± 0.4	5.6 ± 0.5	2.0 ± 0.2
*per 50 g of available carbohydrates*			
Proteins	11.7	12.8	5.1
Carbohydrates (available)	50.0	50.0	50.0
Sugars	8.4	10.6	15.4
Total fat	13.1	15.1	9.8
Total dietary fibers	3.8	4.9	1.4

Values are presented as mean ± SD

**Table 2:** Amino acid composition of the three tested biscuits (BCAAsB, ArgB and CB) expressed in g per 100 g (a) and in g per 50 g of available carbohydrates (b)

Amino Acid	BCAAsB	ArgB	CB
(a)			
L-Alanine	0.691 ± 0.097	0.541 ± 0.076	0.213 ± 0.030
L-Arginine	0.747 ± 0.105	1.050 ± 0.150	0.241 ± 0.034
L-Aspartic acid	1.160 ± 0.160	1.000 ± 0.140	0.298 ± 0.042
L-Cysteine	0.184 ± 0.026	0.235 ± 0.033	0.150 ± 0.021
L-Glutamic acid	3.000 ± 0.420	3.370 ± 0.470	2.250 ± 0.320
Glycine	0.488 ± 0.068	0.587 ± 0.082	0.249 ± 0.035
L-Histidine	0.309 ± 0.043	0.292 ± 0.041	0.145 ± 0.020
Hydroxy-L-Proline	n.d	n.d	n.d
L-Isoleucine	0.547 ± 0.077	0.494 ± 0.069	0.232 ± 0.032
L-Leucine	1.130 ± 0.160	0.938 ± 0.131	0.464 ± 0.065
L-Lysine	0.482 ± 0.067	0.345 ± 0.048	0.123 ± 0.017
L-Methionine	0.181 ± 0.025	0.255 ± 0.036	0.103 ± 0.014
L-Phenylalanine	0.708 ± 0.099	0.674 ± 0.094	0.329 ± 0.046
L-Proline	0.888 ± 0.124	0.911 ± 0.128	0.749 ± 0.105
L-Serine	0.745 ± 0.104	0.655 ± 0.092	0.333 ± 0.047
L-Threonine	0.479 ± 0.067	0.444 ± 0.062	0.190 ± 0.027
L-Tryptophan	0.148 ± 0.015	0.179 ± 0.018	0.076 ± 0.008
L-Tyrosine	0.396 ± 0.055	0.401 ± 0.056	0.185 ± 0.026
L-Valine	0.637 ± 0.089	0.615 ± 0.086	0.288 ± 0.040
L-Ornithine	n.d	n.d	n.d

Amino Acid	BCAAsB	ArgB	CB
(b)			
L-Alanine	0.576	0.476	0.149
L-Arginine	0.623	0.924	0.168
L-Aspartic acid	0.967	0.880	0.208
L-Cysteine	0.154	0.207	0.105
L-Glutamic acid	2.502	2.966	1.573
Glycine	0.407	0.517	0.174
L-Histidine	0.258	0.257	0.101
Hydroxy-L-Proline	n.d	n.d	n.d
L-Isoleucine	0.456	0.435	0.162
L-Leucine	0.942	0.825	0.324
L-Lysine	0.402	0.304	0.086
L-Methionine	0.151	0.224	0.072
L-Phenylalanine	0.590	0.593	0.230
L-Proline	0.741	0.802	0.524
L-Serine	0.621	0.576	0.233
L-Threonine	0.399	0.391	0.133
L-Tryptophan	0.123	0.158	0.053
L-Tyrosine	0.330	0.353	0.129
L-Valine	0.531	0.541	0.201
L-Ornithine	n.d	n.d	n.d

Values are presented as mean ± SD; n.d., not detected

### Insulin resistance

The study subjects (n = 30) successfully completed all three clinical study sessions, and no adverse effects were recorded. All the anthropometric, clinical and biochemical characteristics of NW and OW subjects are presented in Table 3. Subjects in OW group had higher fasting serum insulin, higher HOMA-IR and compromised insulin secretory function, as defined by HOMA-*β* index. More specifically, HOMA-IR and HOMA-*β* values were 2–3 fold higher in OW compared to NW (*p* < 0.01). Participants in both study groups had normal fasting glucose values and no significant differences were observed between them.

**Table 3:** Anthropometric, clinical and biochemical characteristics of normalweight (n = 15) and overweight/obese subjects (n = 15)

Characteristic	Normalweight (n = 15)	Overweight/obese (n = 15)	*p *value
Sex (male/female)	(7/8)	(8/7)	–
Age (years)	25.5 ± 4.9	27.5 ± 5.6	0.309
Body weight (kg)	66.1 ± 11.8	89.3 ± 9.1	**0.000**
BMI (kg/m^2^)	21.9 ± 1.9	29.5 ± 3.0	**0.000**
Body fat %	20.3 ± 7.1	30.8 ± 9.4	**0.020**
WHR	0.72 ± 0.2	0.85 ± 0.1	**0.019**
SBP (mmHg)	103.9 ± 30.1	115.2 ± 10.1	0.301
DBP (mmHg)	64.2 ± 19.4	71.4 ± 8.7	0.426
Glucose (mg/dL)	89.0 ± 5.6	91.7 ± 4.2	0.143
Insulin (μU/mL)	4.9 ± 2.5	11.1 ± 6.9	**0.003**
HOMA-IR	1.09 ± 0.6	2.6 ± 1.5	**0.001**
HOMA-*β*	72.0 ± 30.2	139.7 ± 66.4	**0.001**

BMI, body mass index; WHR, waist to hip ratio; SBP, systolic blood pressure; DBP, diastolic blood pressure; HOMA-IR, homeostasis model assessment for insulin resistance index; HOMA-*β*, homeostasis model assessment for *β*-cell function index. Values are presented as mean ± SD. Numbers in bold indicate significant differences between the two groups at a significance level of *p* < 0.05

### Fasting plasma AAs concentrations

Twenty-four AAs were determined i.e., L-alanine, L-arginine, L-asparagine, L-aspartic acid, L-glutamic acid, L-glutamine, glycine, L-histidine, hydroxy-L-proline, L-isoleucine, L-leucine, L-lysine, L-methionine, L-phenylalanine, L-proline, L-serine, L-threonine, L-tryptophan, L-tyrosine, L-valine, D,L-alpha-aminobutyric acid, L-citrulline, L-ornithine and taurine. On Table 4, fasting plasma AAs concentrations of the two groups of subjects are presented. It is observed that the concentrations of methionine, tryptophan and tyrosine were significantly higher in OW compared to NW subjects (*p* < 0.05, Table 4) with a similar trend in isoleucine concentrations (*p* = 0.053). In contrast, the OW group presented significantly lower fasting plasma levels of glutamine (*p* < 0.05, Table 4) with a similar trend in glycine (*p* = 0.059) and alanine concentrations (*p* = 0.090).

**Table 4:** Fasting plasma amino acids concentrations of normalweight (n = 15) and overweight/obese subjects (n = 15)

Amino acid (μmol/L)	Normalweight (n = 15)	Overweight/obese (n = 15)	*p *value
L-Alanine	268.96 ± 9.83	245.40 ± 8.47	0.090
L-Arginine	69.89 ± 3.68	68.91 ± 2.25	0.828
L-Asparagine	49.71 ± 1.88	47.19 ± 1.32	0.298
L-Aspartic acid	7.22 ± 1.18	6.90 ± 0.29	0.801
L-Glutamic acid	70.12 ± 6.07	76.24 ± 7.92	0.559
L-Glutamine	527.48 ± 18.26	445.33 ± 13.66	**0.002**
Glycine	224.07 ± 8.67	204.40 ± 4.22	0.059
L-Histidine	80.32 ± 2.97	76.83 ± 1.63	0.328
Hydroxy-L-Proline	11.43 ± 0.95	12.62 ± 1.15	0.447
L-Isoleucine	54.27 ± 2.53	61.72 ± 2.52	0.053
L-Leucine	115.24 ± 4.84	115.67 ± 3.80	0.946
L-Lysine	137.20 ± 6.70	137.97 ± 5.51	0.760
L-Methionine	22.12 ± 0.93	27.05 ± 0.92	**0.001**
L-Phenylalanine	50.64 ± 1.71	53.50 ± 1.12	0.186
L-Proline	155.22 ± 9.58	165.02 ± 11.80	0.538
L-Serine	110.41 ± 6.70	100.21 ± 2.59	0.181
L-Threonine	126.44 ± 5.63	117.92 ± 3.87	0.238
L-Tryptophan	54.30 ± 1.94	59.24 ± 1.18	**0.044**
L-Tyrosine	53.49 ± 1.79	59.50 ± 1.27	**0.013**
L-Valine	222.84 ± 6.70	225.65 ± 6.98	0.781
D, L-alpha-Aminobutyric acid	21.94 ± 1.42	20.61 ± 0.89	0.450
L-Citrulline	32.22 ± 2.01	29.06 ± 1.36	0.218
L-Ornithine	50.70 ± 2.35	56.35 ± 2.80	0.147
Taurine	65.02 ± 4.11	71.78 ± 4.18	0.275

Values are presented as mean ± SEM. Numbers in bold indicate significant differences between the two groups of subjects at a significance level of *p* < 0.05

### Postprandial plasma AAs responses

**Fig. 1:**
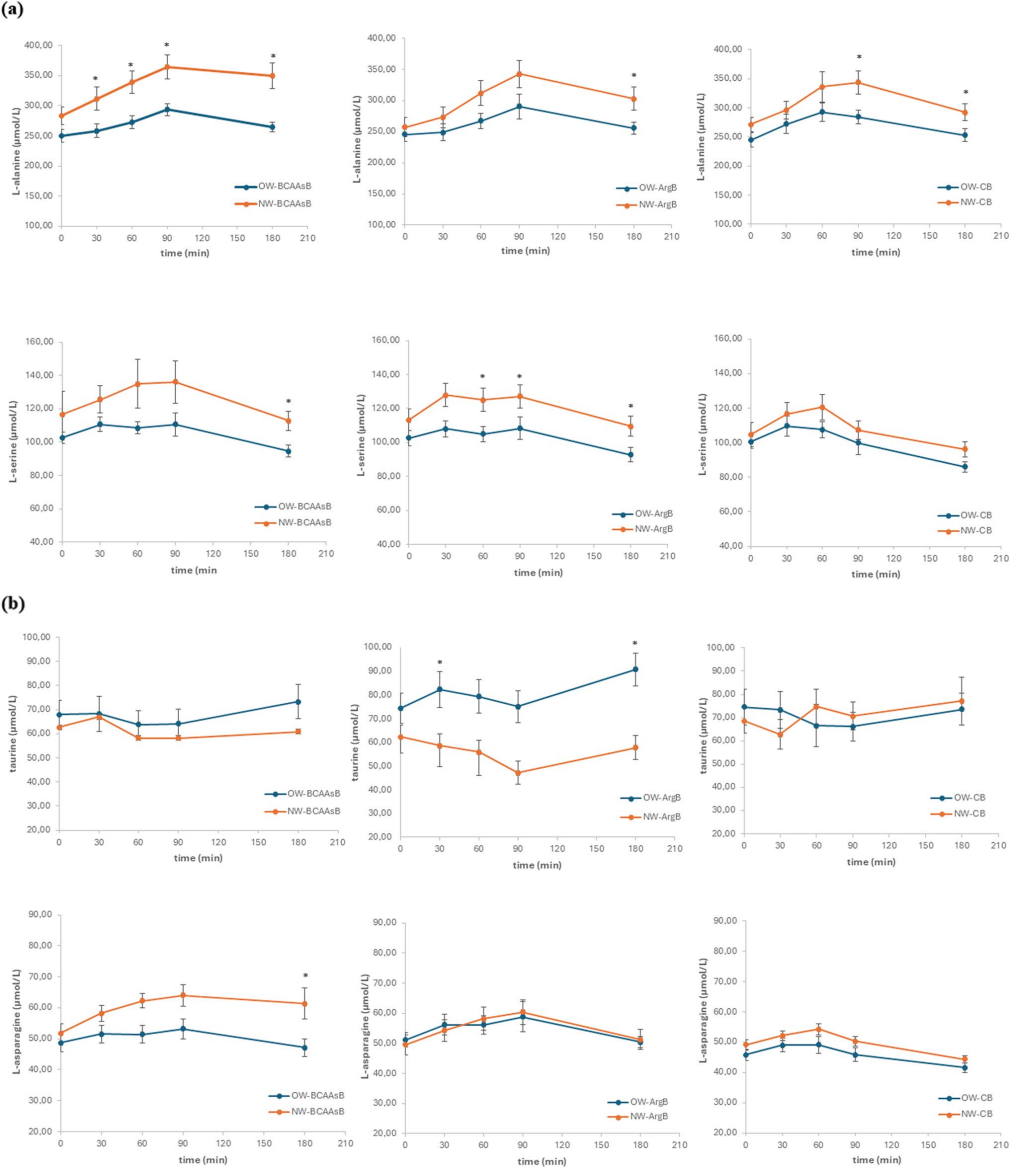

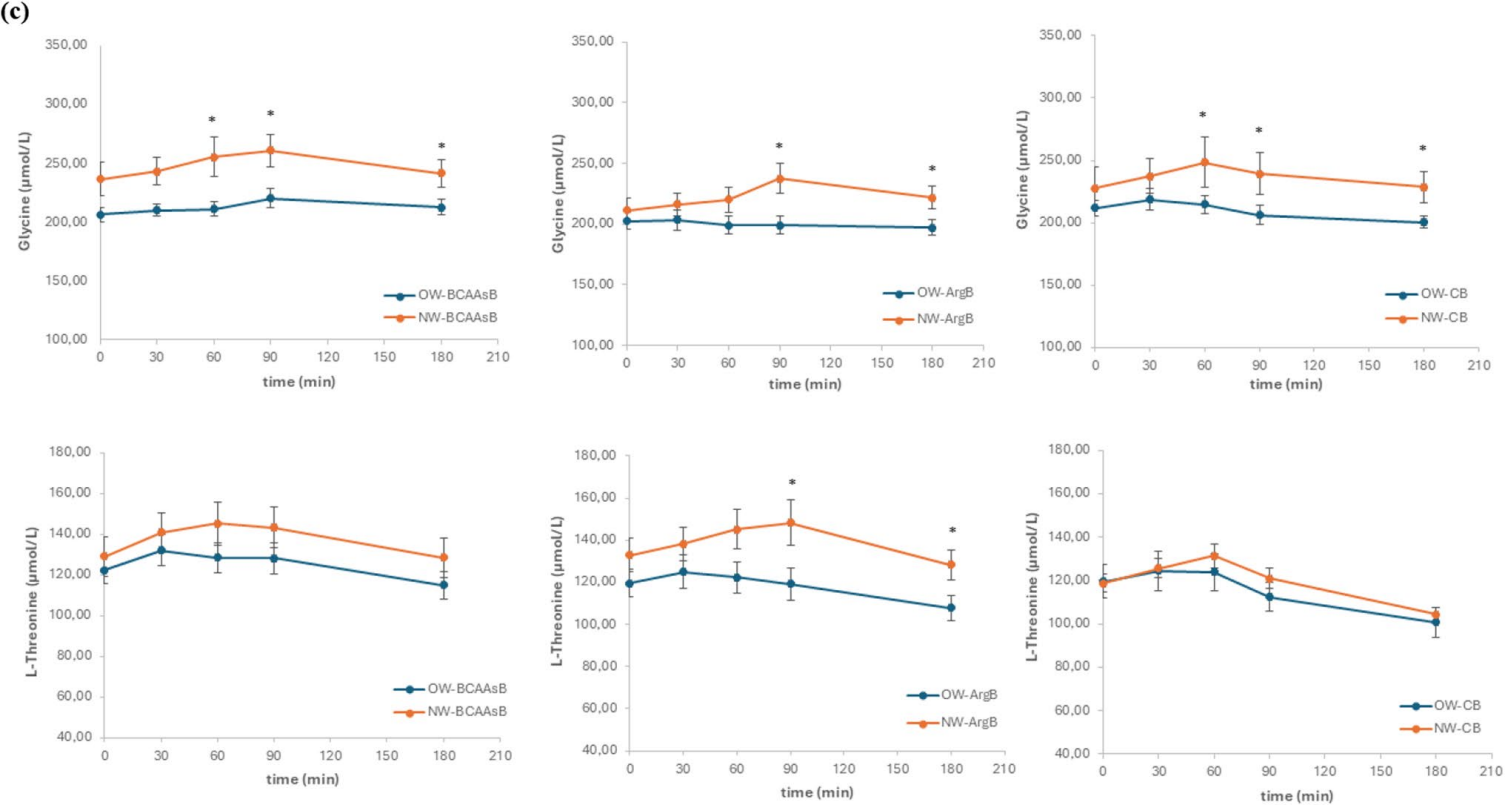
Postprandial responses of L-alanine and L-serine (**a**), taurine and L-asparagine (**b**), glycine and L-threonine (**c**), after consumption of the three biscuits (BCAAsB, ArgB and CB) with different AAs composition, by normalweight subjects (NW) and subjects with overweight/obesity (OW). Values are expressed as mean ± SEM, *p < 0.05 between groups at the same time point

The postprandial changes of AAs concentrations in plasma were measured in three different sessions, after the ingestion of each of the three tested biscuits. As it was expected, the changes after the ingestion of the protein-enriched biscuits were more pronounced compared to those caused by the common wheat biscuit. iAUC values were higher in NW compared to OW subjects independently of the type of biscuit which was consumed (Τable 5, Fig. 1). Significantly lower values in the iAUCs of OW compared to NW group were found for alanine, asparagine, glutamine serine and threonine after ingestion of BCAAsB (*p* < 0.05). After the ingestion of ArgB significantly lower were the iAUCs of glutamine, glycine and threonine (*p* < 0.05) as well as that of alanine (*p* = 0.050). Of note, taurine iAUC was significantly higher in OW subjects (*p* < 0.05). After the ingestion of CB, the concentrations of hydroxyproline, methionine and ornithine were significantly lower in OW group (*p* < 0.05).

## Discussion

Protein metabolism is dysregulated in individuals with metabolic disturbances such as obesity or T2DM. These individuals typically have increased fasting levels of various AAs, including BCAAs and AAAs [6–8, 11, 12, 15]. Other affected AAs with higher concentrations include alanine, glutamic acid and proline, while glycine, serine and glutamine levels are generally lower. These changes are attributed to fat deposition, especially visceral fat accumulation [16, 18] and IR, which promotes muscle protein breakdown and impacts liver gluconeogenesis [8, 9, 38]. Many overweight or moderately obese individuals with normal blood glucose levels are not diagnosed as prediabetics and for this reason they commonly show a delay to proceed to dietary and lifestyle interventions that could prevent metabolic disease progression. The altered AAs metabolism before T2DM diagnosis may predict diabetes onset up to 10 years in advance, especially in those with high visceral fat [9, 12].

In the present study, fasting concentrations of 24 AAs, as well as their postprandial levels at 5 time points following the consumption of plant protein-enriched wheat biscuits with different AAs compositions, were measured in plasma samples from NW and OW subjects. Subjects were apparently healthy, with no apparent underlying conditions other than a BMI > 25 kg/m^2^ in the overweight/obese group. Health status was confirmed through the assessment of basic biochemical parameters, including fasting glucose, insulin, lipid profile, and liver function indices. Significant differences between the two study groups (NW and OW) were observed for a number of AAs, both in fasting as well as in the postprandial state. In the fasting state, serum concentrations of methionine, tryptophan, and tyrosine were significantly higher in OW compared to NW subjects. Significantly higher fasting concentrations of these AAs in obese subjects have been also recorded in other studies [13, 15, 40]. Fasting isoleucine concentrations were higher in OW compared to NW subjects with a *p* value approaching statistical significance (*p* = 0.053); a larger sample size may have revealed a significant difference, as suggested by findings in similar studies [12, 13, 15]. It has been shown that isoleucine, methionine and AAAs have a significant positive correlation with visceral fat as well as subcutaneous fat area [13]. Elevated circulating BCAAs levels have been associated with incomplete oxidation of fatty acids and glucose, potentially contributing to disrupted glucose homeostasis [18, 41]. It has been previously shown that AAAs transport in cells is impaired by the chronic elevations in BCAAs in subjects with overweight/obesity [15].

NW individuals had significantly higher fasting values of glutamine (*p* < 0.05) as well as higher values of glycine compared to OW individuals, for which although the difference did not reach statistical significance (*p* = 0.059), this trend aligns with findings from previous studies comparing similar populations[14, 15]. Higher levels of glutamine, glycine and serine have been also recorded in other studies and have been negatively correlated with body fat and specifically visceral fat, due to the enhancement of oxidation of free fatty acids in the adipose tissue [13, 18, 24]. Decrease in plasma glycine concentration has been correlated with higher visceral fat and insulin resistance [12, 14]. Elevated levels of BCAAs, which are common among obese individuals, lead in glycine depletion and strategies targeting in lower BCAAs concentrations can help to restore glycine levels [11, 24].

In this study the postprandial AAs responses of NW and OW subjects following the ingestion of two biscuits differing in their amino acid composition were evaluated. Significant differences were observed between the two groups in the postprandial responses of certain AAs, as measured by the iAUC. Overall, higher postprandial AAs responses were found in NW, compared to the OW group. More specifically, in the OW group, iAUC, after the ingestion of BCAAsB was significantly lower for alanine, asparagine, glutamine, serine and threonine compared to NW. Regarding the ArgB, significantly lower postprandial responses were found for alanine, glutamine, glycine and threonine, in OW group, whereas iAUC of taurine was significantly higher in this group. For the CB, although the changes were less pronounced compared to the protein enriched biscuits in both study groups, significant differences were observed for hydroxyproline, methionine and ornithine. Similarly with the other two biscuit samples, the NW group had significantly higher iAUCs, compared to OW.

Alanine, glutamine and threonine exerted significantly lower iAUC in OW subjects after ingestion of both plant protein-enriched biscuits (BCAAsB and ArgB). Alanine is a key amino acid involved in hepatic gluconeogenesis due to its ability to convert to pyruvate [11]. As a key gluconeogenic precursor, a greater increase in postprandial alanine provides the body with more capacity to generate glucose, thereby reducing the risk of hypoglycemia [23]. Regarding glutamine, it has been linked with increased circulating levels of GLP-1 and GIP postprandially in both NW and OW individuals. This suggests that glutamine helps in suppressing postprandial glycemia through stimulation of insulin secretion [42, 43]. Individuals with normal body weight seem to more effectively extract and absorb glutamine from the same food product, and through its action on glucose and appetite regulation could contribute to enhanced satiety and thus aid in long-term weight management [12]. Threonine is an essential amino acid that, along with aspartic acid and methionine, aid in fat digestion by the liver, reducing fat accumulation and supporting liver function. Threonine is a precursor of serine and glycine and thus indirectly enhances insulin sensitivity and blood glucose regulation [11, 44].

Lower postprandial glycine levels for all three biscuits were observed in OW individuals. Glycine has been strongly correlated with insulin secretion and interacts closely with BCAAs metabolism, with its circulating levels being inversely associated with BMI [24, 45]. The alteration of glycine response has been also observed in individuals with obesity and is linked with higher risk of T2DM development. According to Alves et al., [11] an explanation for the significantly lower postprandial glycine levels can be a decreased intestinal absorption of glycine in subjects with overweight/obesity. It has been proposed that elevated BCAAs levels in obesity may contribute to glycine depletion, as the body attempts to detoxify excess ammonia (NH₃) produced during BCAAs transamination. This process is often impaired in individuals with obesity, leading to further metabolic disruption. The efficient BCAAs transamination supports proper protein utilization and prevents the accumulation of BCAAs in circulation—a condition often observed in obesity and metabolic disorders. In contrast, impaired transamination can lead to elevated plasma BCAAs levels, which are associated with IR, mitochondrial dysfunction, and altered metabolic signaling [11, 24, 46–48].

Regarding the taurine, a higher postprandial response was observed in OW subjects after the ingestion of ArgB. This may be attributed to the higher ingested amounts of cysteine and methionine through this biscuit, which are precursors of taurine. Taurine is an amino acid which is involved in many biological and physiological functions in the body. It has been shown that in the condition of obesity the levels of taurine are depleted. Supplementation of taurine leads to increased plasma taurine and adiponectin levels in humans while reducing inflammatory and oxidative markers [49].

After consumption of BCAAsB a significantly lower postprandial serine response was observed in OW individuals compared to NW. Higher levels of available serine have been linked with enhanced insulin sensitivity [18]. Regarding asparagine, its postprandial circulating levels were also found to be significantly lower in the OW group. Asparagine is another glucogenic amino acid, with its by-product oxaloacetate used in the tricarboxylic acid cycle for glucose production. Asparagine plays a crucial role in cell catabolism and antioxidant capacity [18].

Considering the findings of the present study alongside relevant literature, individuals with overweight/obesity may absorb and utilize AAs less efficiently than their lean counterparts [7]. As a result, subjects with overweight/obesity are expected to have a lower availability of AAs, which are mainly responsible for the stimulation of protein synthesis (anabolic response), along with the minimization of catabolic response in the body postprandially [9, 50]. Lean individuals generally exhibited more pronounced postprandial responses for most of the AAs measured, even in the cases that their iAUC values were not significantly different from those with overweight/obesity. This consistent trend may indicate physiological differences between groups that could become statistically significant with larger sample size. The small sample size—typical of acute postprandial crossover studies—may limit the power to detect smaller but biologically relevant differences (Table 5). According to the available literature, larger differences between pre- and postprandial amino acid concentrations after ingesting a mixed meal have been correlated with greater IR [51]. In the OW group of subjects significantly higher values of fasting serum insulin, HOMA-IR and HOMA-*β* indexes were present [15].

**Table 5:** Incremental area under the curve (iAUC) of postprandial amino acids responses to the three biscuits (BCAAsB, ArgB and CB) of normalweight (NW, n = 15) and overweight/obese subjects (OW, n = 15)

Amino acid	Group	BCAAsB	*p* value	ArgB	*p *value	CB	*p* value
L-Alanine	NW	9976.9 ± 1577.6	**0.018**	13,773.2 ± 4236.7	**0.050**	7098.4 ± 1122.9	0.192
	OW	4869.3 ± 1279.2		4762.2 ± 1204.2		5300.4 ± 739.2	
L-Arginine	NW	2184.9 ± 300.7	0.587	2840.3 ± 304.8	0.110	811.0 ± 287.2	0.843
	OW	1903.4 ± 420.7		2074.4 ± 352.4		902.0 ± 356.6	
L-Asparagine	NW	1668.1 ± 161.4	**0.000**	1173.4 ± 201.6	0.080	467.4 ± 148.0	0.465
	OW	730.9 ± 157.7		705.4 ± 160.7		324.5 ± 123.5	
L-Aspartic acid	NW	216.4 ± 61.1	0.086	91.4 ± 9.8	0.082	91.4 ± 23.1	0.640
	OW	97.6 ± 27.2		220.9 ± 71.2		75.7 ± 23.8	
L-Glutamic acid	NW	841.8 ± 322.2	0.983	1358.0 ± 463.4	0.810	716.5 ± 266.9	0.189
	OW	850.5 ± 254.6		1508.6 ± 412.1		1749.5 ± 720.1	
L-Glutamine	NW	9346.8 ± 1957.3	**0.044**	6361.9 ± 872.1	**0.004**	7308.5 ± 2036.5	0.293
	OW	4524.3 ± 1192.2		2676.9 ± 794.0		4598.0 ± 1499.1	
Glycine	NW	4242.6 ± 1345.7	0.167	2382.7 ± 620.5	**0.045**	1604.8 ± 449.0	0.248
	OW	2112.6 ± 661.5		950.2 ± 282.7		939.5 ± 340.9	
L-Histidine	NW	1662.4 ± 223.0	0.069	1052.9 ± 220.3	0.652	884.0 ± 179.4	0.087
	OW	1021.1 ± 254.6		1523.0 ± 1007.9		424.7 ± 186.4	
Hydroxy-L-Proline	NW	194.0 ± 66.3	0.143	91.0 ± 19.9	0.207	67.0 ± 18.0	**0.026**
	OW	83.2 ± 31.6		54.7 ± 19.8		21.3 ± 7.3	
L-Isoleucine	NW	1066.7 ± 155.9	0.114	752.4 ± 148.7	0.484	306.3 ± 76.5	0.588
	OW	716.7 ± 147.8		609.6 ± 135.8		398.9 ± 150.5	
L-Leucine	NW	1452.7 ± 216.9	0.625	1000.7 ± 196.1	0.852	435.8 ± 123.1	0.377
	OW	1275.5 ± 285.7		1064.9 ± 278.0		842.1 ± 435.4	
L-Lysine	NW	2558.9 ± 722.7	0.275	1268.0 ± 723.0	0.611	573.7 ± 233.9	0.884
	OW	1612.2 ± 447.6		831.9 ± 444.7		638.1 ± 371.6	
L-Methionine	NW	413.0 ± 129.8	0.112	304.8 ± 100.6	0.418	270.2 ± 68.2	**0.034**
	OW	174.3 ± 66.1		204.5 ± 69.0		102.0 ± 32.8	
L-Phenylalanine	NW	897.4 ± 201.7	0.977	783.6 ± 145.6	0.562	569.4 ± 183.9	0.103
	OW	889.9 ± 160.4		670.3 ± 126.8		240.1 ± 65.6	
L-Proline	NW	3677.4 ± 632.7	0.759	3844.0 ± 593.1	0.612	4342.1 ± 556.4	0.309
	OW	4065.6 ± 1080.3		5200.0 ± 2573.1		3375.6 ± 748.2	
L-Serine	NW	2344.6 ± 405.8	**0.006**	1636.1 ± 276.7	0.099	1304.1 ± 271.1	0.219
	OW	887.4 ± 268.8		976.8 ± 268.9		832.4 ± 259.1	
L-Threonine	NW	2397.7 ± 497.9	**0.046**	1600.8 ± 383.8	**0.022**	811.4 ± 189.1	0.740
	OW	1101.8 ± 368.7		582.6 ± 172.3		1008.3 ± 556.0	
L-Tryptophan	NW	654.0 ± 185.5	0.068	526.3 ± 176.8	0.448	418.3 ± 132.9	0.429
	OW	244.9 ± 110		366.9 ± 107.7		256.8 ± 151.4	
L-Tyrosine	NW	583.0 ± 113.0	0.115	402.1 ± 187.5	0.073	304.2 ± 119.9	0.566
	OW	320.0 ± 115.3		1237.0 ± 407.6		215.2 ± 95.3	
L-Valine	NW	2642.0 ± 515.1	0.187	2008.5 ± 398.8	0.103	890.5 ± 328.4	0.645
	OW	1687.3 ± 439.9		1209.1 ± 257.3		1285.8 ± 781.5	
D, L-alpha-Aminobutyric acid	NW	414.4 ± 150.9	0.064	112.4 ± 36.00	0.432	139.9 ± 37.1	0.940
	OW	114.8 ± 37.7		77.1 ± 25.8		146.8 ± 83.8	
L-Citrulline	NW	227.2 ± 151.1	0.422	66.1 ± 30.8	0.558	131.1 ± 84.9	0.222
	OW	97.4 ± 50.1		45.9 ± 14.7		519.0 ± 298.6	
L-Orthithine	NW	1544.2 ± 342.8	0.821	1335.3 ± 309.4	0.755	758.0 ± 173.5	**0.010**
	OW	1445.4 ± 262.5		1463.0 ± 261.9		234.3 ± 73.2	
Taurine	NW	922.0 ± 470.8	0.478	670.0 ± 226.2	**0.040**	1038.1 ± 248.6	0.927
	OW	1547.0 ± 731.0		2060.0 ± 603.1		998.7 ± 349.0	

NW, normalweight; OW, overweight. Values are presented as mean ± SEM. Numbers in bold indicate significant differences between the two groups (normalweight and overweight/obese) at a significance level of *p* < 0.05

Specific AAs could act as metabolic signals involved in appetite regulation, either through the stimulation of gut-derived satiety hormones or via central mechanisms. Table 6 provides an overview of AAs physiological role and proposed mechanisms of action, highlighting their relevance in postprandial metabolism. It is assumed that generally higher postprandial concentrations of several AAs which was observed in the NW group may contribute to more effective signaling for appetite control. This trend is consistent with previous literature suggesting a greater sensitivity to nutrient signaling in lean individuals [47, 52–54]. For instance, the iAUC of the glutamine, which was significantly higher in the NW group for both protein-enriched biscuits, has been associated with increased GLP-1 secretion, a gut peptide secreted by enteroendocrine cells as AAs and small peptides enter in the small intestine. The elevated postprandial GLP-1 levels have been associated with slower rate of gastric emptying, enhanced satiety and reduced hunger [42, 43, 55]. The postprandial threonine response following the ingestion of both BCAAs-enriched and L-arg-enriched biscuits was significantly lower in the OW group compared to the NW group. Elevated postprandial circulating threonine levels have been associated with increased satiety and reduced subsequent ad libitum food intake. These findings underscore the complexity of protein-induced satiety, which may rely on indirect and complex signaling pathways rather than a direct action of circulating AAs on the brain [54, 56]. In individuals with overweight or obesity, this attenuated amino acid response may contribute to reduced gut hormone secretion and impaired central signaling, potentially leading to impaired appetite regulation. As a result, this population may face greater challenges in effectively managing body weight due to decreased sensitivity to postprandial metabolic signals [47].

**Table 6:** Overview of single amino acids and potential actions regarding obesity and T2DM

Amino acid	Key functions/actions	Associations with obesity/T2DM	Potential mechanisms
Branched-Chain amino acids (Leucine, Isoleucine, Valine)	Regulate glucose, lipid, and energy metabolism, are mainly metabolized in muscle [18]	Elevated levels are linked to: Higher fasting insulin, HOMA-IR and HbA1 (increased risk of T2DM development) Higher BMI and visceral fat (obesity presence) [12, 15, 19, 47]	Leucine activates the key signaling systems within hypothalamic neurons (mammalian targets of rapamycin/AMP activated protein kinase, mTOR/AMPK) that regulate glucose metabolism Suppresses food intake through regulation of neuropeptide Y/Agouti-related peptide (NPY/AgRP) and proopiomelanocortin (POMC) release [28–30]
Aromatic amino acids-Phenylalanine	Involved in protein synthesis and neurotransmitter production	Elevated levels are associated with peripheral IR and higher risk for T2DM [18, 57]	Modifies insulin receptor beta (IRβ) subunit and inhibits insulin signaling and glucose uptake by muscle and adipose tissues
Aromatic amino acids-Tyrosine	Involved in glucose transport into the cells and gluconeogenesis	Higher levels are linked with poor glycemic regulation and IR and higher BMI and WC Early biomarker for T2DM [12, 15, 58]	Superfluous tyrosine can be rapidly catabolized and impair clearance of circulating glucose and increase gluconeogenesis [18]
Aromatic amino acids-Tryptophan	Precursor for serotonin and kynurenine pathway [18]	Lower circulating levels have been correlated with higher fasting insulin, triglycerides, blood pressure and inflammation Higher risk forT2DM and obesity [58, 59]	Elevated levels of certain tryptophan-derived metabolites due to increased activity of kynurenine pathway that promotes inflammation and metabolic stress Disruption in serotonin pathway increases appetite and promotes weight gain [60]
Glutamate	Produced endogenously via the conversion of asparagine to aspartate, followed by transamination to glutamate Precursor for GABA and a major neurotransmitter in central nervous system [61]	Higher levels are observed in obesity and have been linked with IR and higher risk for T2DM [61, 62]	Affects central nervous system signaling involved in appetite control, by inhibition of insulin receptor/Akt/mTOR pathway that leads to an increase in energy intake [62]
Alanine	Primary gluconeogenic substrate in the liver that links muscle to liver metabolism	Elevated levels in obesity contribute to impaired glucose metabolism and IR [43]	Increased alanine levels lead to elevated hepatic gluconeogenesis and excessive glucose production from the liver
Methionine	Precursor for the methyl donor (SAM) that is involved in DNA methylation and lipid metabolism [63]	Elevated levels in obesity Higher levels are linked to impaired glucose tolerance and *β*-cell function [13]	Elevated levels lead to increased homocysteine and lead to oxidative stress and endothelial disfunction Altered lipid and glucose metabolism that leads to fat accumulation and IR [15, 40, 63]
Glutamine	Appetite regulation and promotion of gut health [42]	Lower circulating levels are linked to increased levels of body fat [12, 18]	Increases the secretion of the GLP-1, stimulates insulin secretion and reduces postprandial glycaemia and enhances satiety [43]
Glycine	Precursor for glutathione synthesis and availability resist damage caused by oxidative stress [18]	Lower plasma concentrations correlated with higher BMI, HOMA-IR, and visceral fat [12, 14, 64]	Most closely linked amino acid to insulin sensitivity Elevated levels of BCAAs lead to glycine depletion [11, 24]
Asparagine	Involved in protein synthesis, antioxidant ability and cell catabolism and signaling [18]	Low levels are associated with prediabetes and hyperglycemia [58]	Involvement in the AMPK-mTORC1 pathway that regulates cellular energy balance [20]
Serine	Supports insulin secretion and glucose homeostasis and can be synthesized from glycine [65]	Deficiency is linked to obesity, IR, *β*-cell dysfunction [13, 65]	Insufficient levels of serine lead to abnormal synthesis of phospholipids and the accumulation of deoxysphingolipids that contribute to pancreatic cell dysfunction [66]
Threonine	Supports liver fat metabolism Precursor of glycine and serine Protection of gut barrier integrity [11]	Lower circulating levels of threonine have been recorded in obese population [67]	Enhancing lipid metabolism by reducing fat buildup in the liver Indirectly enhances insulin sensitivity Beneficial impact on gut microbiota and metabolic signaling linked with appetite and energy balance [67]
Arginine	Improvement of endothelial function Enhancement of glucose metabolism Reduction of appetite and food intake [30, 32]	Lower concentrations of arginine have been correlated with obesity and IR	Endogenous precursor of nitric oxide synthesis Promotion of insulin mediated glucose uptake by increasing blood flow Increase of GLP-1 and PYY circulating levels [31, 68, 69]

The results of the present study showed that subjects with overweight/obesity have lower, less pronounced AAs responses after the consumption of the same plant protein-based snack compared to normalweight and different AAs composition causes different postprandial AAs responses which can further affect metabolic dysregulation in the condition of overweight/obesity. However, it must be mentioned that plant protein structures differ from animal proteins due to different peptide sequences, leading to distinct secondary and tertiary structures and consequently different digestibility and functional properties [70, 71].

A limitation of the present study is the relatively small sample size with only 15 participants in each group, occurred after further analysis of existing obtained samples. This constraint limits the robustness of the conclusions and reduces the possibility of detecting statistically significant differences across the examined parameters. However, it must be noted that this is a demanding postprandial protocol and it is difficult to recruit a large number of participants. Another important limitation that may have influenced the outcomes of the present study is the level of participants’ compliance with the study protocol. Specifically, participants were required to adhere to standardized guidelines on the evening prior to each test day and throughout the entire experimental period (i.e., between study sessions). Variations in adherence to these instructions, including dietary intake, physical activity, or other lifestyle factors, may have introduced variability in the postprandial responses observed.

These findings suggest that the amino acid composition of foods influences postprandial amino acid metabolism differently in NW and OW individuals. This highlights the potential for further research in food science towards the development of functional foods with specific amino acid profiles as part of dietary strategies for weight management supporting improvements in metabolic outcomes such as insulin sensitivity, body composition and circulating amino acid profiles.

## Conclusion

In conclusion, beyond their established physiological and nutritional roles as essential or complementary AAs, the functional properties of specific AAs, such as metabolic signaling and appetite regulation should also be considered for the development of functional foods targeted for individuals with overweight or obesity. Given the altered postprandial responses and metabolic dysregulation often observed in this population group, tailoring amino acid composition in food formulation may support more effective appetite control and weight management. These foods, designed with a focus on their amino acid composition, could be effectively incorporated into both balanced and restrictive dietary plans. In balanced diets, they may support overall nutritional adequacy while enhancing satiety and metabolic regulation. In more restrictive dietary interventions—such as those aiming for energy reduction or macronutrient control—they can serve as functional components that help preserve lean body mass, regulate appetite, and potentially improve postprandial metabolic responses. These findings highlight the importance of moving beyond simply protein enrichment and considering the specific amino acid profile, which may offer additional metabolic benefits, particularly for individuals with overweight or obesity.

## Data Availability

The data that support the findings of this study are available from the corresponding author upon reasonable request.
